# Lights off - Role of bioluminescence for the biology of the biocontrol agent *Photorhabdus luminescens*

**DOI:** 10.1016/j.isci.2024.110977

**Published:** 2024-09-17

**Authors:** Friederike Pisarz, Luca Rabbachin, Fabio Platz, Alice Regaiolo, Ralf Heermann

**Affiliations:** 1 Johannes Gutenberg University Mainz, Institute of Molecular Physiology, Microbiology and Biotechnology, Hanns-Dieter-Hüsch-Weg 17, 55128 Mainz, Germany; 2 Institute for Biotechnology and Drug Research gGmbH (IBWF), Hanns-Dieter-Hüsch-Weg 17, 55128 Mainz, Germany

**Keywords:** Biological sciences, Plant biology, Interaction of plants with organisms, Plant pathology

## Abstract

Bioluminescence is found across various organisms having crucial functions for biotic interactions and stress adaptation. The only known terrestrial bioluminescent bacteria are entomopathogenic bacteria of the genus *Photorhabdus*. However, the reason why these bacteria produce light is not understood. *P. luminescens* exists in two cell forms called primary (1°) and secondary (2°) cells. The 1° cells colonize the nematode symbiosis partner and produce bright light, whereas 2° cells colonize plant roots only emitting weak light. Here we show that bioluminescence is important but not essential for the biology of the bacteria. Deletion of the *luxCDABE* operon in 1° cells impaired insect pathogenicity and nematode interaction. The complete loss of light of 2° cells resulted in enhanced plant root colonization, enhanced haemolysis, and reduced oxidative stress adaptation. Since bioluminescence is not essential for the survival of the bacteria, *P. luminescens* Δ*lux* 1° and 2° emerged as useful tools for bioluminescence-based reporter assays.

## Highlights

• Inactivation of the *luxCDABDE* operon in *P. luminescens* results in a dark phenotype
• Bioluminescence impacts biotic interactions and stress response of *P. luminescens*
• *P. luminescens* Δ*lux* mutants can be used for bioluminescence-based reporter assays

## Graphical abstract

**Figure:**
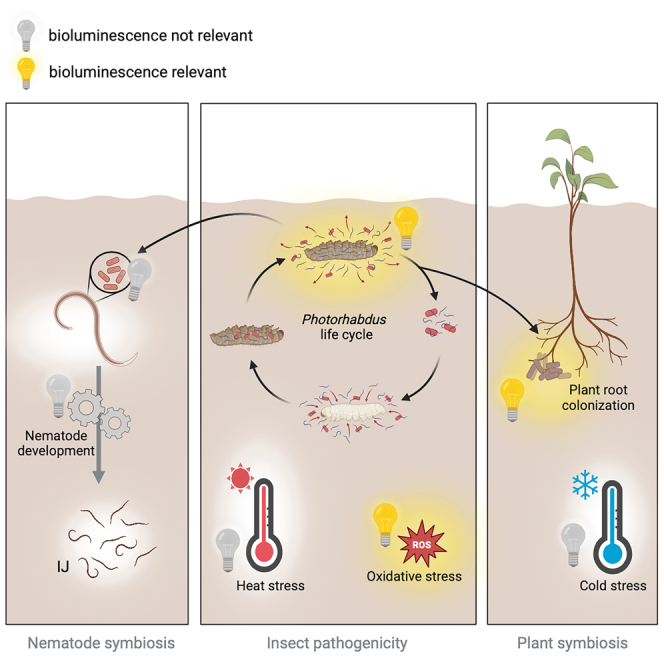

## Introduction

Bacterial bioluminescence has attracted much attention since its first discovery. During the chemical reaction light is emitted, whereby the luciferase LuxAB catalyses the oxidation from FMNH_2_ under the consumption of oxygen.^1^ To date, all known bioluminescent bacteria are Gram-negative and most are found in a marine environment.^2^ The biological functions can reach from defense, prey attraction, communication to counterillumination or symbiosis with i.e., fish or squids.^3^^,^^4^^,^^5^ However, the terrestrial bacterium *Photorhabdus luminescens* is also bioluminescent due to expression of the *luxCDABE* operon.^6^ Whilst in marine bacteria such as *Vibrio fischeri* bioluminescence plays an important role for establishing bacterial-host interactions and is relevant for the formation of the squid light organ.^7^ Yet, even though many studies have been performed, no direct role for bioluminescence could be assigned for *P. luminescens*, the only terrestrial bacterium found to date deploying bioluminescence.^8^^,^^9^ *Photorhabdus luminescens* subsp. *luminescens* strain DJC is a Gram-negative entomopathogenic bacterium characterized by insect pathogenicity, plant protection, phenotypic heterogeneity, and name-giving bioluminescence.^10^^,^^11^^,^^12^ The two phenotypic different cell forms are designated as primary (1°) and secondary (2°) cells. The 1° cells live in mutualistic symbiosis with nematodes of the genus *Heterorhabditidae bacteriophora* and are highly pathogenic toward insects.^13^ In addition, there are other properties well-known to *P. luminescens* 1°, such as the production of stilbene and anthraquinones.^14^^,^^15^ All these phenotypic traits including bioluminescence are highly repressed or absent from 2° cells, respectively. In contrast, 2° cells can colonize and protect plant roots through the production of a chitinase against phytopathogens such as *Fusarium graminearum*.^10^^,^^16^

The role of bioluminescence for the physiology and ecology of *P*. *luminescens* is not yet understood. Previous studies have shown low bioluminescence in *P. luminescens* 1° cells in the exponential growth phase, but very high in the late logarithmic growth phase.^17^ However, no potential reason could be defined why bioluminescence decreases in the stationary phase. One of the Patterson’s hypotheses is the attraction of uninfected larvae to an already infected larva, which reduces the distance of the nematodes to a new host and therefore supports the nematode-bacteria symbiosis.^18^ Another hypothesis suggests that bioluminescence is an event of horizontal gene transfer.^8^ Furthermore, it has been suggested that bioluminescence in *P. luminescens* has a simple physiological importance, i.e., reducing the oxidizing conditions in the insect hemolymph when the bacteria reach their high growth rates after infection using the oxygen-dependent luciferase.^19^ According to Clarke, dark mutants of *P. temperata* are not able to support nematode development and therefore stated, bioluminescence underlies environmental selection.^20^ However, the role of bioluminescence in *P. luminescens* DJC has not been investigated yet, and the importance of the biology of the bacteria is unclear. Yet, the complexity of the *P. luminescens* life cycle, involving multiple eukaryotic interactions, makes the bacterium an interesting candidate as a biocontrol agent. Therefore, extensive studies are essential to determine the various aspects of *P. luminescens* on e.g., the role of the bioluminescence, the microflora of plants, and the optimal environmental conditions to exploit its full potential. Reporter assays, using fluorescent proteins or bioluminescence, are one way of investigating gene expression or cellular events of bacteria.^21^ While fluorescent proteins have high stability, indicating gene activity, the long half-time of fluorophores cannot reflect accurately the on- and off-phase of the respective genes.^22^ Furthermore, a high fluorescence background is often detected depending on the autofluorescence of media compounds or different secondary metabolites produced by the bacteria.^14^ In contrast, luminescence-based reporter assays are more sensitive and enable the study of gene activity in bacteria during symbiosis with other organisms without external interfering factors or false positives.^23^ Thus, the *P. luminescens luxCDABE* operon has been used for more than 20 years to monitor gene expression in Gram-negative bacteria.^14^ To date, luminescence-based reporter assays could not be performed in *P. luminescens* due to its intrinsic luminescence. Therefore, we aimed to shed light on the role of bioluminescence in *P. luminescens* and further establish a luminescence-based reporter assay. Thereby, we generated a *P. luminescens* DJC Δ*lux* mutant in 1° as well as in 2° cells and analyzed various phenotypes that are important for the life cycle of the bacteria. As light production was not essential for growth of *P. luminescens* in both cell variants, we demonstrate that bioluminescence can therefore be perfectly used as a reporter in the engineered *P. luminescens* DJC Δ*lux* 1° and 2° variants.

## Results

### The role of bioluminescence in the growth of *P. luminescens*

**Figure 1:**
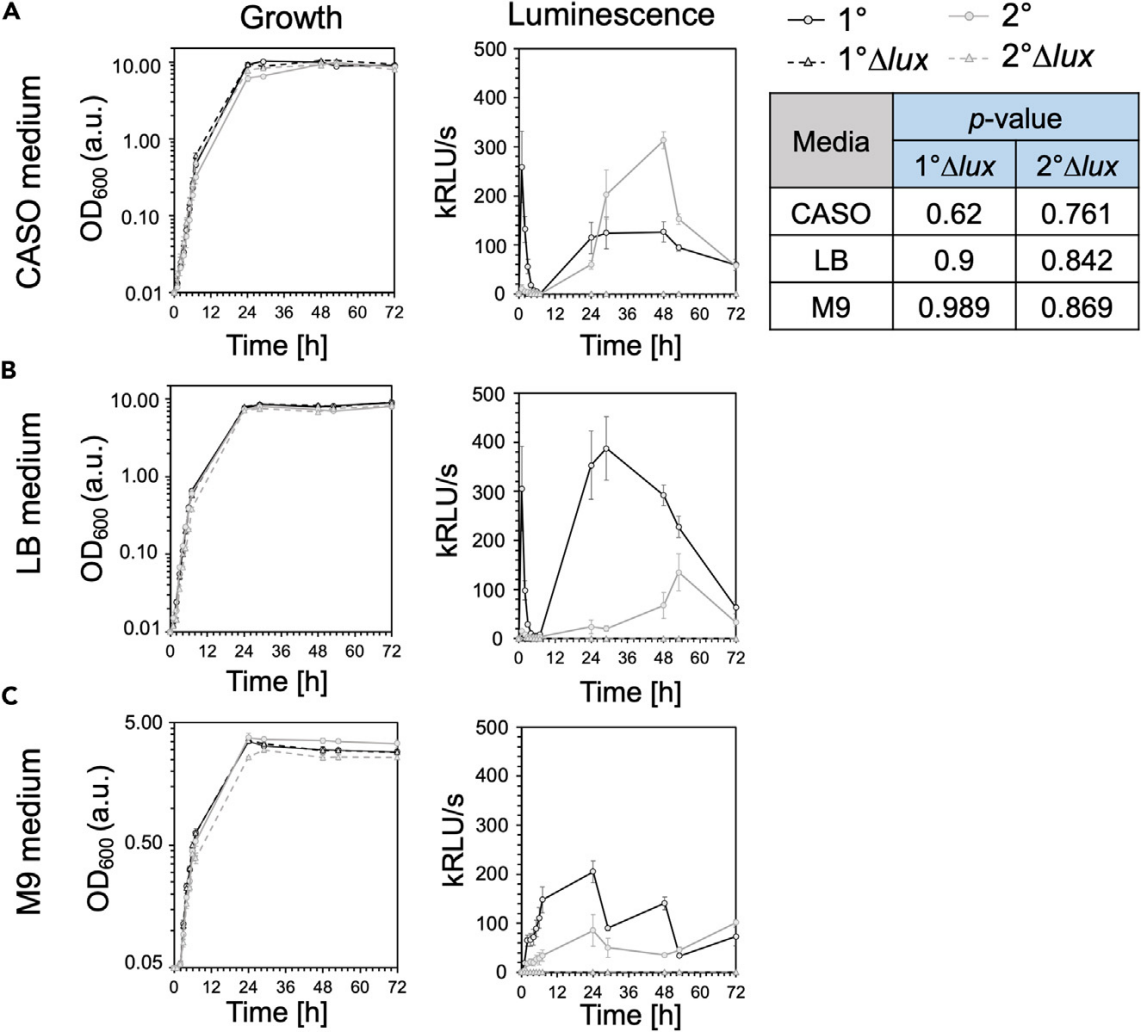
Growth and bioluminescence of *P. luminescens* DJC wildtype and Δ*lux* mutants Growth was monitored over 3 days in CASO medium (A), LB medium (B) and M9 medium (C). Y axis shows the OD_600_ representing logarithmic bacterial growth or the luminescence as relative luminescence (kRLU/s) and the X axis the time in hours. Values are means of three independent biological replicates; error bars represent the standard deviation. The *p*-value using linear regression of OD_600_ was calculated to examine growth differences of the different strains in the same media.

To determine whether bioluminescence plays a functional role in *P. luminescens* DJC such as its growth as well as its adaptation to various environmental changes, we generated mutant strains of *P. luminescens* strain DJC 1° and 2° cells lacking the complete *lux* operon (genes *luxCDABE*; *PluDJC_11100* – *PluDJC_11120*). These mutants are further referred to as Δ*lux.* In the initial phase of the study, growth tests were carried out by comparing the growth rate of the *P. luminescens* wildtype 1° and 2° variants compared to the isogenic Δ*lux* mutants in different culture media (Figure 1). No significant differences were observed in bacterial growth when cultivated in either CASO medium (Figure 1A), LB medium (Figure 1B) or M9 medium (Figure 1C). As expected, no luminescence was detected in the *P. luminescens* Δ*lux* strains, whereas a high luminescence was detected in *P. luminescens* 1° cells in CASO or LB medium, after 2 h of cultivation and in the stationary phase. In summary, the deletion of the *lux* operon did not impair bacterial growth and therefore these results showed no correlation between the absence of bioluminescence and bacterial growth when cultivated under standard laboratory conditions.

### The role of bioluminescence for stress response and enzymatic activities in *P. luminescens*

**Figure 2:**
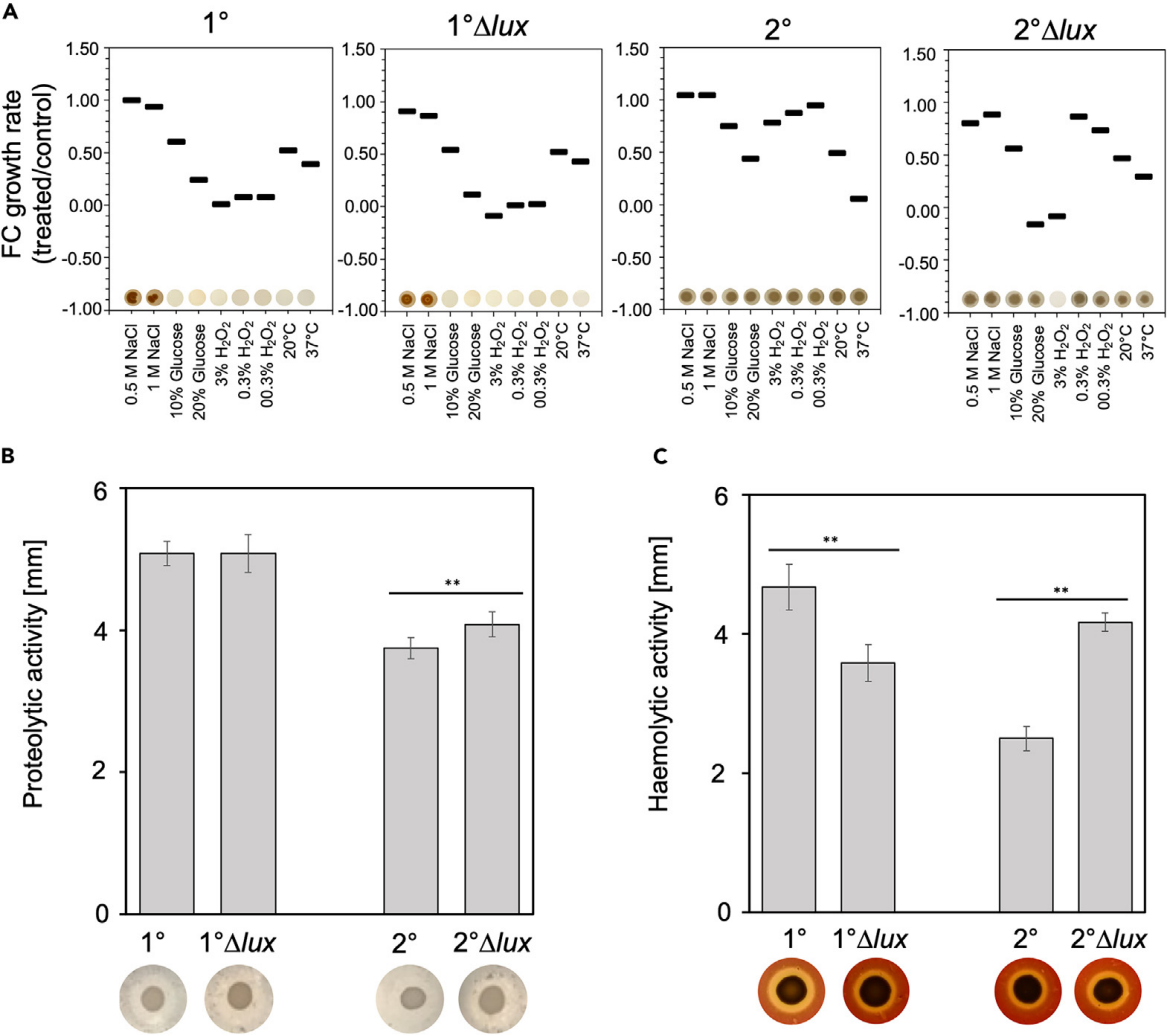
Phenotypical characterization of the *P. luminescens* DJC 1° and 2° wildtype and the respective Δ*lux* strains (A) Bacterial growth and ability to form colonies tested under different stress conditions. Bacterial growth was measured for 3 h after stress induction, and the growth rate (μ) was calculated in the linear growth phase within the 3 h after stress treatment or without treatment in the same time frame. Displayed is the Fold-change of the treated group against the control group. Colony formation on solid media after stress induction is indicated in the pictures below. (B) Analysis of proteolytic activity on caseinate agar plates after 2 days. (C) Secreted hemolytic activity of wildtype and Δ*lux* strains on sheep blood agar plates after 5 days. Errors bars represent the standard deviation of three independently performed experiments. Representative pictures of one trial are displayed. Statistical significance (*p* ≤ 0.05) was calculated using a t-test and is indicated with ∗∗.

We further investigated whether the lack of bioluminescence affects enzymatic activities such as proteolysis or hemolysis or whether it plays a crucial role in stress adaptation mechanisms, which are important for the biology of *P. luminescens*. Therefore, as the second step the growth rate and colony development of the *P. luminescens* Δ*lux* 1° and 2° cells were evaluated in comparison to the respective wildtype. Accordingly, we exposed the bacteria to osmotic salt and sugar stress, oxidative stress and different temperatures measured the OD_600,_ and monitored their ability to form colony forming units. Thereby, we could identify possible correlations between bioluminescence and the bacterial response to environmental stresses (Figure 2A). Surprisingly, the *P. luminescens* 1°Δ*lux* mutant showed a similar fold change of growth rates compared to the isogenic wildtype, however, colony growth was absent in both *P. luminescens* 1° and 1°Δ*lux* cells under most conditions besides salt stress, where colony formation was observed. In contrast, the *P. luminescens* 2°Δ*lux* strain showed a significant decrease in growth and was no longer able to form colonies after the addition of 3% (v/v) H_2_O_2_, while the *P. luminescens* 2° wildtype was still able to form colonies. Nonetheless, no distinctive pattern could be identified in which the deletion of the *lux* operon plays a crucial role in stress-coping in both, *P. luminescens* 1° and 2° cells.

Next, to test the proteolytic and hemolytic activity semi-quantitative phenotypic assays were performed. For that purpose, the bacteria were plated on caseinate and sheep blood agar, respectively. No significant change in proteolytic activity was observed for the Δ*lux* strains compared to the respective wild type. Only a slight increase in proteolysis was observed for the *P. luminescens* 2°Δ*lux* cells (Figure 2B). However, a significant shift was observed for hemolytic activity in both, *P. luminescens* 1°Δ*lux* and 2°Δ*lux* cells. While a decrease of haemolysis was observed for the *P. luminescens* 1°Δ*lux* strain compared to its wildtype, a significant increase was observed for the *P. luminescens* 2°Δ*lux* cells, with an almost 50% increase in hemolytic activity (Figure 2C). In summary, the absence of bioluminescence due to the deletion of the *lux* operon in the *P. luminescens* 1° cell form did not affect the adaptation to stress as well as the tested enzymatic activity. However, the increased hemolysis observed in *P. luminescens* 2°Δ*lux* cells was accompanied by an impaired ability to adapt to high oxidative stress, suggesting a correlation of bioluminescence with the underlying mechanisms.

### Influence of bioluminescence on the interaction of *P. luminescens* with eukaryotic hosts

We then addressed our focus on a comprehensive investigation of the relevance of bioluminescence in the context of the interactions of *P. luminescens* DJC with its eukaryotic hosts that are important at different stages of their life cycle. For that purpose, we investigated insect pathogenicity, nematode colonization as well as plant root colonization of *P. luminescens* DJC 1° as well as 2° cells and the respective mutants lacking their ability to produce light.

**Figure 3:**
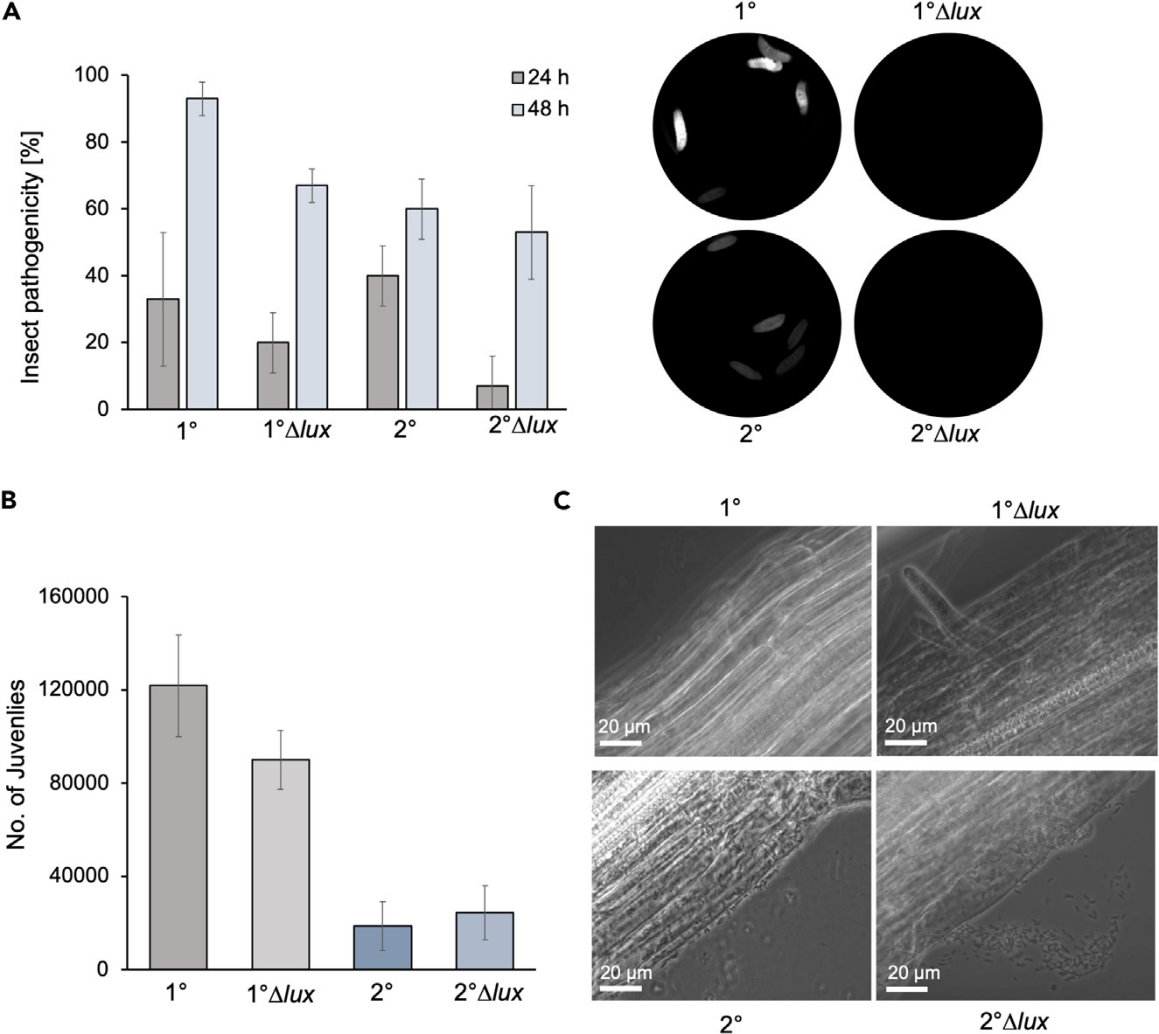
Bacteria-host interactions of *P. luminescens* 1° and 2° Δ*lux* strains (A) Insect pathogenicity of *P. luminescens* toward *Galleria mellonella* larvae. Five larvae were injected with 2.000 cells and mortality was observed over 48 h. Larvae are shown 24 after infection with 2.000 cells of the respective strains. Luminescence was detected with the Bio-Rad Gel Doc XR^+^ Gel Documentation System. (B) Nematode bioassays are performed with *Heterorhabditis bacteriophora* on the respective *P. luminescens* wildtype and Δ*lux* strains. Number of infected juveniles after 21 days is shown. (C) Phase contrast microscopy of *Arabidopsis thaliana* roots after 14 days of co-cultivation with *P. luminescens* wildtype and Δ*lux* strains. The pictures represent one characteristic of three independent biological replicates. Errors bars represent the standard deviation of three independently performed experiments. Statistical significance is indicated by the t-test (∗∗, *p* ≤ 0.05).

First, the insect pathogenicity of *P. luminescens* wildtype and Δ*lux* strains was determined by infecting *Galleria mellonella* larvae by injecting 2.000 cells of the respective bacteria, and mortality was monitored over 48 h (Figure 3A). The *P. luminescens* 1° wildtype showed a lower pathogenicity toward the insect larvae, the mortality of the larvae in the presence of the bacteria was reduced to 35% after 24 h, whereas *P. luminescens* 1°Δ*lux* pathogenicity was reduced by 20%. A reduction in mortality of the larvae was also observed for the *P. luminescens* 2°Δ*lux* strain, with a 30% reduction in mortality of the insect larvae. Additionally, the infected insect larvae were monitored 24 h post-infection regarding bioluminescence produced by *P. luminescens,* and indeed, luminescent larvae, dead or alive, were observed after infection with *P. luminescens* 1° and 2° wildtype, but not with the respective Δ*lux* cells variants. However, after 48 h the pathogenicity was restored in the *P. luminescens* 2°Δ*lux* cells, while the pathogenicity of the *P. luminescens* 1°Δ*lux* cells was still decreased by 25%. This indicates that the bioluminescence could play a role in the insecticidal activity of *P. luminescens* after insect infection as the loss of bioluminescence led to a reduced insect larvae mortality in the first stages after infection.

Then, we tested whether the *P. luminescens* 1°Δ*lux* cells were still able to re-associate with nematodes and if *P. luminescens* 2° cells were still unable to engage in nematode symbiosis. For that purpose, we counted the number of infective juveniles (IJs) emerging after 21 days of feeding of 50 hermaphrodites on the respective bacterial strain. Approximately 120.000 developed after co-cultivation with the *P. luminescens* 1° wildtype with the hermaphrodites. A slight decrease of 25% IJs was developed compared to the wildtype (Figure 3B). As expected, a significantly lower number of IJs (approximately 20.000) developed on the *P. luminescens* 2° cells. However, the number of IJs developed on the *P. luminescens* 2°Δ*lux* strain was comparable to the wild type. In conclusion, bioluminescence is important for nematode symbiosis of *P. luminescens*, however, is not essential for successful nematode reproduction and development as the luminescent deficient strain was still able to promote nematode development.

It could be demonstrated before that *P. luminescens* 2° cells specifically colonize plant roots.^16^ Although bioluminescence is strictly reduced in 2° cells, we nevertheless tested the role of the remaining light of *P. luminescens* for plant interaction by analyzing the colonization of *P. luminescens* DJC 2° Δ*lux* strains with *Arabidopsis thaliana*. For that purpose, *A*. *thaliana* Col-0 seedlings were co-cultivated with the respective strains for 14 days. Then, the colonization of the roots by the presence of surrounding bacteria or those saddled on the roots were analyzed by microscopy (Figure 3C). As expected, *P. luminescens* 2° wildtype cells could be detected accumulating to the plant roots as well as in the surroundings of the roots. However, the accumulation of cells was also observed with *P. luminescens* 2°Δ*lux* cells, whereby an increased number of cells attached to the roots became visible when microscopically analyzed. This analysis indicates a positive effect on the interaction of *P. luminescens* Δ*lux* 2° cells with plant roots compared to the wild type.

In summary, the deletion of the *luxCDABE* operon and therefore the loss of bioluminescence had slight effects on the *P. luminescens*-host interactions, however, it was not essential for the interaction with the different eukaryotic hosts under the conditions tested. While the *P. luminescens* 1°Δ*lux* strain is impaired to promote nematode development and reduced insect pathogenicity, the *P. luminescens* 2°Δ*lux* strain showed an enhanced colonization of plant roots, suggesting a less crucial role of the bioluminescence in the *P. luminescens* 2° cells than for the 1° cells.

### Using the *lux* operon for bio luminescence-based reporter assays in *P. luminescens*

**Figure 4:**
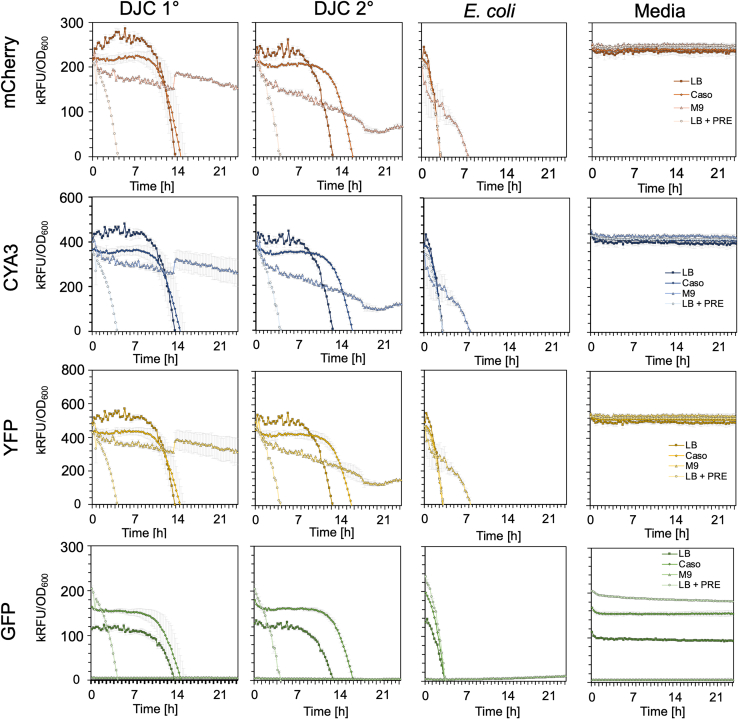
Fluorescence background detected in *P. luminescens* 1° and 2° cells and *E. coli* DH5α λ*pir* *P. luminescens* 1° and 2° cells, as well as E. coli, were cultivated in LB, CASO, M9, and LB + 3% PRE at 30°C for 24 h, and OD_600_ as the fluorescence was measured (GFP = excitation/emission 485/535 nm, YFP = excitation/emission 513/530 nm, CYA3 = excitation/emission 579/591 nm, mCherry = excitation/emission 587/610 nm). The respective growth media served as blank controls. The normalized fluorescence with the OD_600_ was plotted against the time. Three biological replicates were performed, and error bars represent the standard deviation of three biological trials.

**Figure 5:**
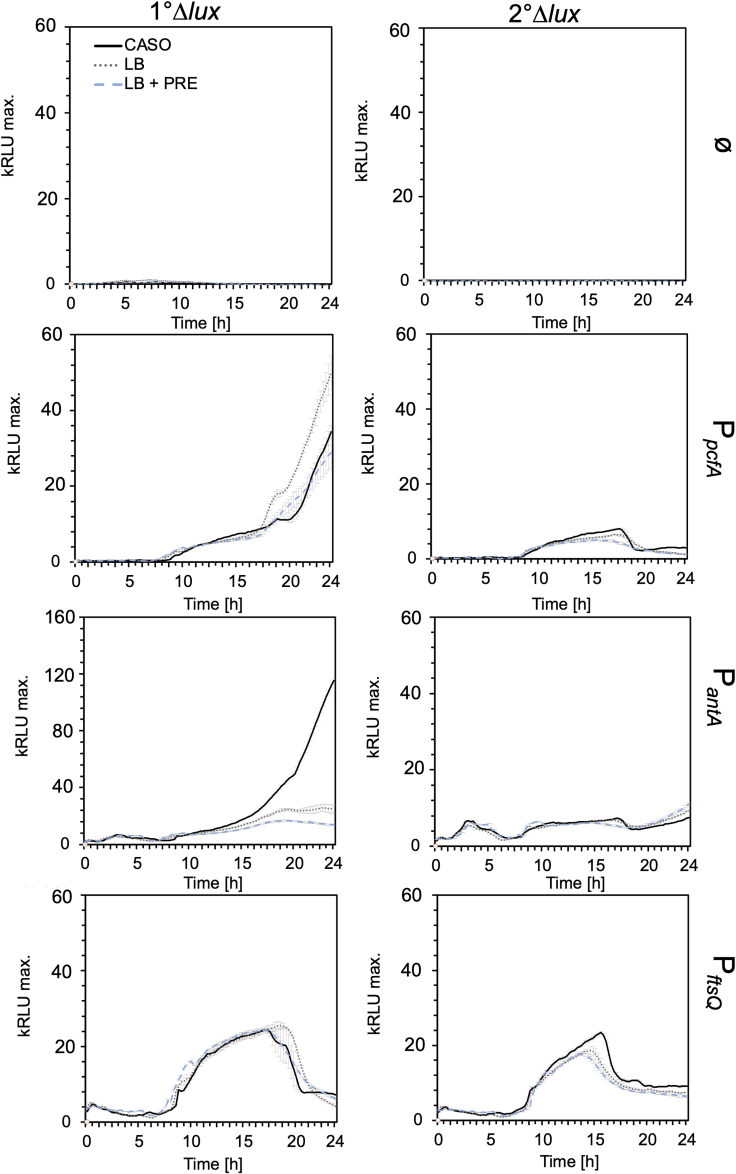
Bioluminescence-based reporter assays in *P. luminescens* *P. luminescens* 1°Δ*lux* and 2°Δ*lux* carrying the reporter plasmids pBBR1-luxP_*pfcA*_, pBBR1-luxP_*antA*_ and pBBR1-luxP_*ftsQ*_ were cultivated in CASO, LB, and LB medium with or without the addition of 3% plant root exudates (PRE) for 24 h. OD_600_ and luminescence were measured in a microplate reader. The y axis indicates the relative luminescence and the x axis the time in hours. *P. luminescens* 1°Δ*lux* and 2°Δ*lux* carrying the empty pBBR1-lux plasmid served as control (ø). Errors bars represent the standard deviation of three independently performed experiments.

Fluorescence-based reporter assays are commonly used to detect the activity of bacterial promoters monitoring gene expression. However, the production of secondary metabolites by *P. luminescens* as well as the culture medium can cause background noise and therefore generate false positive results. Since bioluminescence was not observed to be essential for the biology of *P. luminescens*, we tested whether the *P. luminescens* DJC Δ*lux* 1° and 2° cells could be used for bioluminescence-based reporter assays. First, we tested the background signal representing the fluorescence channels used for the detection of various fluorophores (e.g., mCherry, CYA3, YFP, GFP), representing the condition if no expression of these fluorophores is present in the cells. In *P. luminescens* DJC, background noises due to e.g., secondary metabolite production were detected over a time span of 24 h with significant variations in signal strength depending on the culture media, whereas background noises detected for *E. coli* are significantly less (Figure 4). To monitor gene regulation *in vivo* in *P. luminescens*, it is therefore more suitable to apply luminescence-based reporter assays, as no background noise was observed in the initial growth tests performed of the *P. luminescens* 1° and 2°Δ*lux* strains (Figure 1). Therefore, a luminescence-based reporter assay can improve the precision and lower false positives due to the high background fluorescence of *P. luminescens* while examining promoter activities *in vitro*. First, we transformed *P. luminescens* DJC 1°Δ*lux* and 2°Δ*lux* with the pBBR1-*lux* plasmid carrying the *P. luminescens luxCDABE* operon under the control of the *pcfA*, *antA,* or *ftsQ* promoter regions, respectively, and measured bioluminescence in different growth phases of the bacteria over 24 h (Figure 5). While the promoter of *pcfA* and *antA* have already been shown to be active in the exponential phase in *P. luminescens* 1° cells, activity of the *ftsQ* promoter should be detected in the exponential growth phase, as the protein is crucial for cell division.^24^^,^^25^^,^^26^^,^^27^ The empty reporter plasmid served as control to rule out the possibility of background luminescence. While no bioluminescence could be detected in the controls, activity of the *pcfA* promoter in the *P. luminescens* 1° cells was observed in the late exponential phase, followed by a significant increase after 20 h in the stationary phase. Similar to the promoter activity of *pcfA*, the *antA* promoter activity increased with the stationary phase in *P. luminescens* 1°Δ*lux* cells but not in *P. luminescens* 2°Δ*lux* cells. However, a significant difference between the culture media could be observed, where the activity of the *antA* promoter was significantly higher in CASO than in the LB medium. In contrast, the *ftsQ* promoter was active in the exponential growth phase in both *P. luminescens* 1°Δ*lux* and 2°Δ*lux* cells and not in the stationary growth phase. In summary, we could observe the expected promoter activities of the different reporter strains as expected from previous results. Furthermore, these results show that the deletion of the *luxCDABE* operon can be complemented in 1° as well as in 2° cells *in trans*. In conclusion, we could highlight that a bioluminescence-based reporter assay is functional in *P. luminescens* and is, therefore, a versatile tool for investigating promoter activities alternatively to the less-sensitive and fluorescence-based reporter assays.

## Discussion

Bioluminescence, a phenomenon found in various kingdoms attracted much research since its first observations. Bacterial bioluminescence is mostly found in marine bacteria, where it plays a role in symbiosis with marine fish or squids. However, bacteria of the *Photorhabdus* species live in terrestrial habitats and no role of bioluminescence has been assigned yet.^14^^,^^28^ With our study, we aimed to clarify the role of the bioluminescence in the insect pathogen *P. luminescens* DJC, an entomopathogenic bacterium previously proposed as a very useful biocontrol agent due to its high bioinsecticide activity as well as plant-growth promoting and antifungal properties.^10^^,^^16^ Thereby, we were able to generate *P. luminescens* mutants lacking the *luxCDABE* operon, which harbors the crucial genes encoding bacterial luciferase. First, we could show that the lack of bioluminescence does not affect the vitality of *P. luminescens* DJC, i.e., that the inactivation of the corresponding genes is not lethal for the bacteria. Furthermore, we could show that light production is important for the interaction with the eukaryotic hosts, yet not crucial to establish these interactions. Based on these results, the *lux*-mutants became accessible for use as putative bioluminescence-base reporter strains through a plasmid-based reporter system, which can be subsequently used to study the phenotypical heterogeneity at gene expression level in both cell variants, the 1° as well as the 2° cells. This offers a more sensitive approach than a fluorescence-based reporter system due to high background fluorescence probably due to the production of a high subset of secondary metabolites by the cells.

Bioluminescence can consume up to 20% of cell energy, making it a highly energy-intensive process.^29^ To determine whether the lack of bioluminescence promotes bacterial growth, respective growth assays were performed. However, in the absence of bioluminescence, no increase or decrease in bacterial growth was observed. Therefore, we can conclude that bioluminescence is not related to bacterial growth under standard laboratory conditions. Therefore, bioluminescence-based reporter assays can be performed without being affected by negative cell growth. Yet, upon the induction of oxidative stress through the addition of 3% H_2_O_2_, the reduced bacterial growth and the lack of colony formation in *P. luminescens* 2°Δ*lux* cells show that bioluminescence also plays an important physiological role. In the process of bioluminescence, the luciferase LuxAB catalyzes the oxidation of reduced flavin (FMNH_2_) to oxidized flavin (FMN) under the consumption of O_2_ and it was previously suggested, that bioluminescence protects *P. luminescens* against reactive oxygen species, for example under highly oxidizing conditions in insect hemolymph.^28^^,^^30^ However, cell death was only observed in *P. luminescens* 2°Δ*lux* cells at high concentrations of hydroperoxide, as it has also been observed for *Vibrio harveyi* Δ*luxA* and Δ*luxB* mutant, suggesting that bioluminescence plays a supporting but not a vital role in adaptation to oxidative stress.^31^

Further analyses of the *P. luminescens* Δ*lux* mutants showed that the lack of bioluminescence does not influence the proteolytic activity, but hemolytic activity with a significant increase in the *P. luminescens* 2° cells and reduction in the *P. luminescens* 1° cells. Presumably, the lack of bioluminescence in the *P. luminescens* 2°Δ*lux* cells could provide energy for other cellular processes such as hemolysis. Indeed, an increase in *P. luminescens* 2°Δ*lux* cells attached and close to the plant roots could also be observed, which suggests similar benefits upon bioluminescence absence although bioluminescence is highly repressed in 2° cells compared to 1° wildtype cells. Previous studies suggested that the lack of bioluminescence is a benefit for the bacteria due to the lower energy costs and that dark mutants could outcompete the luminescent strains.^32^^,^^33^ However, studies of O’Grady showed, that dark mutants are rather rare and do not bring evolutionary advantage compared to the wildtypes.^34^ A further argument against this conclusion is the results of the analyses of the *P. luminescens* 1°Δ*lux* mutant, which is impaired in interacting with nematodes, reduced promotion of the reproduction of IJs, and showed a reduced insect pathogenicity. Therefore, we can conclude that the bioluminescence of the 1° cell form of *P. luminescens* is an advantage in the interaction with the eukaryotic hosts, but it is not essential. However, bioluminescence deficiency in *P. luminescens* DJC 1°Δ*lux* appears to be associated with reduced pathogenicity toward insect larvae, probably by a lack of oxidative stress response in the insect cadaver as previously suggested.^35^ Defense mechanisms of the insect larvae could be dependent on the availability of reactive oxygen species, which would be depleted by luciferase activity.^35^ Thus, the absence of luciferase could result in the larvae’s defense mechanism remaining intact, causing reduced pathogenicity of the bacteria.

In conclusion, the absence of bioluminescence had different effects on the different cell forms of *P. luminescens*. While *P. luminescens* 1° cells are impaired in their interaction with their eukaryotic hosts, *P. luminescens* 2° cells benefit from the absence of luminescence, leading to the increased colonization of plant roots and increased hemolysis. The results again highlight the significant differences between *P. luminescens* 1° and 2° cells, which need to be further investigated to enable the future use of *P. luminescens* as a potent biocontrol agent. The application of bacteria as BCA demands studies of various characteristics, including the interaction with other microorganisms. To elucidate unresolved characteristics between the 1° and 2° cell forms, luminescence-based reporter assays can be used to uncover the underlying gene regulatory mechanisms that lead to the colonization of plant roots with 2° cells on the one hand and to a symbiosis of 1° cells and nematodes on the other. We therefore first tested luminescence-based reporter assays as a further step to optimize the analysis of the two distinct phenotypes as fluorescence-based reporter assays are often not suitable in *P. luminescens*. For this purpose, the promoter activity of P_*pcfA*_, P_*antA,*_ and P_*ftsQ*_ was examined over a period of 24 h in different media. The expression of the *pcf* gene cluster leads to cell clumping and increased virulence, especially in the *P. luminescens* 1° cells^25^,^26^. This is also consistent with the results of the *lux*-reporter assays carried out here, where we detected a slight increase in promoter activity in the late exponential phase and a significant increase in the early stationary phase. Furthermore, an altered reporter activity was detected when plant root exudates were added to the medium. Moreover, the activity of P_*antA*_, the promoter for the synthesis of anthraquinone, a pigment present in the 1° cell form of *P. luminescens*,^27^ was found to increase upon entry into the stationary phase in the CASO medium. In contrast, in the *P. luminescens* 2° cells, only P_*ftsQ*_ activity was detected in the exponential growth phase, which is also consistent with the expected activity, as FtsQ is a low abundance protein essential for cell division. While studies on promoter activities have been performed before, we can provide here a more detailed study on the promoter activities *in vivo*, monitoring promoter activities over a longer time period without the influence of culture media or secondary metabolites.

### Conclusion

Here we could shed light on the role of bioluminescence in the biology of *P. luminescens* DJC. In particular, the reduced pathogenicity and impaired interaction with nematodes in the *P. luminescens* 1° cells caused by the incapability of the bacteria to produce light are of interest, as bioluminescence is generally higher in 1° cells and therefore plays a more important role in the life cycle of the 1° compared to the 2° cells. However, a role of bioluminescence could be assigned in the *P. luminescens* 2° cells as well, supporting the role of light production as adaptation mechanism for oxidative stress in the future. Therefore, transcriptome analyses could unravel the link between bioluminescence and oxidative stress by identifying unknown or further investigating known gene regulation pathways in *P. luminescens*. Finally, the re-introduction of the *lux* genes via a plasmid-based created a versatile reporter system that can be used to investigate bacterial-host interactions in depth over a prolonged time in the future. Overall, this work provides further insights into the complex life cycle as well as into the biology of *P. luminescens* and supports understanding the role of bioluminescence in terrestrial bacteria.

### Limitations of the study

Our study demonstrates that the bioluminescence of *P. luminescens* has an impact on the biology of the bacteria, affecting biotic interactions to the three eukaryotic hosts as well as the oxidative stress response. However, it remains unclear whether light emission itself has direct impact on the biology of the bacteria and is sensed by specific receptors, or if light influences other physicochemical parameters within the cell thereby having an indirect effect on the bacterial physiology. It could also be possible that the bacterial light is sensed by the eukaryotic host, i.e., insects, nematodes as well as plants, thereby affecting the biotic interactions with the bacteria. Finally, there are also some limitations for the application of the *P. luminescens* Δ*lux* mutants for bioluminescence-based reporter gene assays. It is not recommended to perform those assays with promoters of genes that are involved in the physiology of the bacteria affected by bioluminescence as those assays are not performed in wildtype backgrounds.

## Resource availability

### Lead contact

Further information and requests for resources should be directed to and will be fulfilled by the lead contact, Ralf Heermann (heermann@uni-mainz.de).

### Material availability

The bacterial strains, plasmids and all new materials described in this study will be shared upon reasonable request from the lead contact.

### Data and code availability

• This article does not report the original code.
• Any data reported in this article is available from the lead contact upon request.
• Any additional information reported in this article is available from the lead contact upon request.

## Acknowledgments

Research was funded by the “Inneruniversitäre Forschungsförderung (“Stufe I“),” 10.13039/501100004033Johannes Gutenberg University of Mainz, Germany, to A.R. We thank Kirsten Schaubruch (JGU) for excellent technical assistance.

## Author contributions

Fr.P and L.R. performed the molecular biological and microbiological experiments and analyzed the data. Fa.P. performed the nematode bioassays. Fr.P. and R.H. designed, and R.H. and A.R. supervised the experiments. Fr.P., A.R., and R.H. wrote the article. All authors participated in the scientific discussion and in writing, read and approved the final version of the article.

## Declaration of interests

The authors declare no competing interests.

## STAR★Methods

### Key resources table

**Table:** 

REAGENT or RESOURCE	SOURCE	IDENTIFIER
**Bacterial and virus strains**		

*Photorhabdus luminescens* subsp. *luminescens* DJC 1°	Zamora-Lagos et al.^36^	DJC 1°
*Photorhabdus luminescens* subsp. *luminescens* DJC 2°	Zamora-Lagos et al.^36^	DJC 2°
*Photorhabdus luminescens* DJC 1°Δ*lux*	This study	1°Δ*lux*
*Photorhabdus luminescens* DJC 2°Δ*lux*	This study	2°Δ*lux*

**Biological samples**		

*Galleria mellonella*	Reared in our lab	N/A
*Arabidopsis thaliana* Col-O	Grown in our lab	N/A
*Heterorhabditis bacteriophora*	Reared in our lab	N/A

**Oligonucleotides**		

CTAACTGCAGTTTGTATATAAAGAAGAGCTTGAT	This study	FA_lux_operon_PstI
CGTCAGTAGATCATTAGCCATCCATTTAATGG	This study	FA_lux_operon_ovl
GATCTACTGACGTATACTCTATGGATTTTAAGATGC	This study	FB_lux_operon_ovl
GACCGGATCCGAACATGAATAAAGTGATACTTCT	This study	FB_lux_operon_BamHI

**Recombinant DNA**		

pNTPs138-R6KT::lux	This study	N/A
pBBR1-*lux*-P_pcfA_	Brachmann et al.^37^	N/A
pBBR1-*lux*-P_*antA*_	Heinrich et al.^27^	N/A
pBBR1-*lux*-P_*ftrsQ*_	Brameyer et al.^38^	N/A

**Software and algorithms**		

CGGC: Compare groups of growth curves	Walter and Eliza Hall Institute of Medical Research	https://bioinf.wehi.edu.au/software/compareCurves/

### Experimental model and study participation details

Key resources table contains a list of all strains used or generated in this study. All tested strains were cultivated in liquid medium at 30°C unless other stated in the method details section. Strains that needed to be preserved were stored in 60% glycerol at −80°C.

*Arabidopsis thaliana* seeds were surface sterilized using 50% bleach (v/v), rinsed with sterile water five times, and sown on MS agar plates (0.4% [w/v] MS Basal Salt moisture, 3% [w/v] Sucrose, 0.8% [w/v] agar). The agar plates were transferred to a growth chamber and incubated at 24°C under a 16-h-light/8-h-dark time period for up to 14 days.

### Method details

#### Bacterial strains and cultivation

*Escherichia coli* strain ST18^39^ were cultivated in LB medium (1% [w/v] tryptone, 0.5% [w/v] yeast extract, 0.5% [w/v] NaCl) at 37°C, shaking under aerobic conditions. *Photorhabdus luminescens* subsp. *luminescens* strain DJC^36^ was aerobically cultivated at 30°C in CASO medium (1.5% [w/v] peptone, 0.5% [w/v] peptone from soy, 0.5% [w/v] NaCl).

#### Generation of plasmids

To generate the plasmid pNTPs138-R6KT::lux, 300 bp upstream (FA) and downstream (FB) of the *lux* operon (*PluDJC_11100* to *PluDJC_11120*) were amplified by PCR using the primer pairs FA_lux_operon_PstI + FA_lux_operon_ovl and FB_lux_operon_ovl + FB_lux_operon_BamHI, introducing a PstI and BamHI restriction side. PCR products were fused by overlap extension PCR and cloned into the pNTPs::138-R6KT plasmid. Correct insertion was confirmed by PCR using the primers check-pNTPs fwd and check-pNTPs-rev, followed by DNA sequencing.

#### Generation of *P. luminescens* mutant and reporter strains

For in-frame deletion strains *P. luminescens* Δ*lux*, conjugation and double homologous recombination was performed as previously described.^40^ Therefore, the respective plasmid was conjugated from *E. coli* strain ST18 into *P. luminescens* 1° and 2° cells and screened for kanamycin resistance. As the pNTPs138-R6KT plasmid contains the *sacB* gene, a second screening was performed to isolate clones with Suc^R^ Km^S^ phenotype. Successful deletion of the *luxCDABE* operon was confirmed by PCR using the primer pair FA_lux_operon_PstI + FB_lux_operon_BamHI, followed by DNA sequencing.

For construction of *P. luminescens* 1° and 2° reporter strains, the respective plasmids pBBR1-*lux*-P_pcfA_,^37^ pBBR1-*lux*-P_*antA*_,^27^ pBBR1-*lux*-P_*ftrsQ*_^38^ and pBBR1-luxø were transferred into *E. coli* ST18 cells via transformation. Plasmids were transferred through conjugation and selection on Gm^R^.

#### Growth assays

To determine whether deletion of the *luxCDABE* affects the cell growth of *P. luminescens*, growth was monitored under different conditions. Therefore, 50 mL of CASO or LB medium were inoculated with the bacteria at OD_600_ = 0.01 and 50 mL of M9 medium was inoculated at OD_600_ = 0.05. Cultures were grown under aerobic conditions at 30°C for 72 h. OD_600_ and luminescence was measured every hour until 7 h and following every 24 h. Three biological replicates were performed. Statistical analyses were performed through linear regression of logarithmically transformed data.

For stress tests, *P. luminescens* cells were grown overnight in CASO medium and following adjusted to OD_600_ = 0.1. At OD_600_ = 1, stress was induced upon adding stock solutions of different solutions to a final concentration of i) 0.5 M or 1 M NaCl, ii) 3%, 0.3% or 0.03% H_2_O_2_ or iii) incubation temperature adjusted to 16°C or 37°C. After further cultivation for 3 h, serial dilutions were spotted on agar plates and growth on solid medium was analyzed. Growth rates were calculated within a 3 h the time after stress treatment. Respective fold changes of the growth rates (μ) treated group versus the control group were calculated to display differences in bacterial growth.

#### Insect pathogenicity assays

Insect pathogenicity assays were performed as previously described.^36^ Larvae of *Galleria mellonella* (reared in our lab) were numbed on ice and surface sterilized with 80% [v/v] ethanol. With a sterile Hamilton syringe (1702 RN, 25 μL, Hamilton), 2 x 10^3^ cells of the *P. luminescens* wildtype or Δ*lux* cells were injected into the hemocoel and the larvae following incubated at 30°C for 48 h. After 24 h and 48 h, mortality was determined, and luminescence visualized (Bio-Rad Gel Doc XR^+^ Gel Documentation System). Sterile medium was injected into the larvae as control, three biological replicates were performed with 5 larvae.

#### Protease bioassays

Semi-quantitative proteolytic activity was determined on caseinate agar plates (1% [w/v] skim milk, 0.3% [w/v] yeast extract, 1.2% [w/v] agar). Briefly, overnight cultures of *P. luminescens* wildtype and Δ*lux* strains were adjusted to OD_600_ = 1 and 50 μL spotted on the mid of a skim-milk agar plate. Plates were incubated for 2 days at 30°C. Three biological replicates were performed.

#### Haemolysis bioassays

Haemolysis was semi-quantitative determined on sheep blood agar (0.5% [w/v] NaCl, 1.0% [w/v] meat extract, 1.0% [w/v] peptone, 0.5% [v/v] sheep blood, 1% [w/v] agar, pH 7.5). Overnight cultures of *P. luminescens* WT and Δ*lux* strains were adjusted to OD_600_ = 1 and 50 μL spotted on the mid of a haemolysis agar plate. Plates were incubated for 4 days at 30°C. Three biological replicates were performed.

#### Plant root colonization assays

To determine the ability of *P. luminescens* to colonize plant roots, a root colonization assay was performed as previously described.^16^ Briefly, *Arabidopsis thaliana* Col-0 seedlings were cultivated on MS agar plates (0.4% [w/v] MS Basal Salt moisture, 3% [w/v] Sucrose, 0.8% [w/v] agar) for 7 days at 24°C with 16 light and 8 h dark regime. *P. luminescens* DJC wildtype and Δ*lux* strains were grown overnight at 30°C and washed with 10 mM MgSO_4_. Then, 120 μL of OD_600_ = 0.02 were spotted on the seedling root tip and visualized by phase-contrast microscopy with the 100x magnitude after 7 and 14 days, respectively (Leica Dmi8 Fluorescence Imaging System). 10 mM MgSO_4_ served as control, three independent biological replicates were performed.

#### Nematode bioassays

To determine the ability of *P. luminescens* to promote the development of *Heterorhabditis bacteriophora*, nematode bioassays were performed. First, 50 μL of *P. luminescens* overnight cultures adjusted to OD_600_ = 1 were spread as a Z onto lipid agar plates (1% [v/v] corn syrup, 0.5% [w/v] yeast extract, 5% [v/v] cod liver oil, 2% [w/v] MgCl2 6xH2O, 2.5% [w/v] Difco nutrient agar [Becton, Dickinson, Heidelberg, Germany]) and incubated for 72 h at 30°C. Then, 50 surface-sterilized axenic *H. bacteriophora* injective juveniles were added and following incubated at RT for up to 21 days. The nematode recovery was monitored after 7 days, 14 days and 18 days by counting the number of infective juveniles (IJs).

#### Extraction of plant root metabolites

Plant root exudates were extracted as previously described.^16^ Briefly, exudates were collected from *Pisum sativum* variant *Arvica,* grown for 2 weeks at 24°C with 16 light and 8 h dark regime in vermiculite. Then, roots of 75 plants were collected and stirred in 250 mL 50% ethanol or 100% methanol for 12 h. Exudates were sterilized through an 0.22 μm sterile-filter and stored at −20°C until further use.

#### Bioluminescence-based reporter assays

To determine bioluminescence as well as background fluorescence in different media, reporter assays were performed in a Tecan Spark microplate reader (Tecan, Salzburg, Austria). Briefly, overnight cultures of *P. luminescens* 1°, 2° and *E. coli* DH5α λ*pir* were washed with 1x PBS-buffer and OD_600_ adjusted to 0.05 in the respective media. Respective media were used as negative control and blank zero points. Then, 200 μL of the cultures were transferred in a black 96-well plate with transparent bottom and incubated for 24 h at 30°C, measuring the OD_600_, luminescence and fluorescence (GFP = excitation/emission 485/535 nm, YFP = excitation/emission 513/530 nm, CYA3 = excitation/emission 579/591 nm, mCherry = excitation/emission 587/610 nm). Values were normalized by using the formula (sample signal – negative control signal)/OD_600_. Three biological replicates were performed.

### Quantification and statistical analysis

Unless otherwise stated, at least three biological independent experiments were performed for each of the assays. Values shown represent the mean ± standard error. Additionally, t-tests were performed to analyze significance (marked with ∗∗). To analyze the significance of bacterial growth curves, linear regression of logarithmically transformed data was performed.
